# A Zn^II^ complex of ornidazole with decreased nitro radical anions that is still highly active on *Entamoeba histolytica*†

**DOI:** 10.1039/d0ra02597f

**Published:** 2020-06-17

**Authors:** Promita Nandy, Soumen Singha, Neha Banyal, Sanjay Kumar, Kasturi Mukhopadhyay, Saurabh Das

**Affiliations:** Department of Chemistry (Inorganic Section), Jadavpur University Kolkata – 700 032 India dasrsv@yahoo.in +91 33 24146223 +91 8902087756; Department of Physics, Jadavpur University Kolkata – 700 032 India; School of Environmental Sciences, Jawaharlal Nehru University New Delhi – 110 067 India

## Abstract

A monomeric complex of Zn^II^ with ornidazole [Zn(Onz)_2_Cl_2_] decreases formation of the nitro-radical anion (R–NO_2_˙^−^), and this is realized by recording it in an enzyme assay using xanthine oxidase, which is a model nitro-reductase. Although the formation of R–NO_2_˙^−^ is essential for drug action, as it is also associated with neurotoxic side effects, it is imperative to control its generation in order to avoid excess presence. With a decrease in R–NO_2_˙^−^, while the neurotoxic side effects should decrease, it can be expected that a compromise with regard to therapeutic efficacy will be seen since the complex will be less active in the free radical pathway. Since R–NO_2_˙^−^ is crucial for the functioning of 5-nitroimidazoles, we attempted to find out if its biological activity is affected in any way in our effort to control its formation. For this purpose, *Entamoeba histolytica* (HM1:IMS Strain) was chosen as a biological target to realize the performance of the complex with respect to ornidazole (R–NO_2_). The experiments revealed that the complex not only compares well with ornidazole, but in fact, under longer exposure times, it also performs better than it. This efficacy of the complex was seen despite a decrease in R–NO_2_˙^−^, as identified by an enzyme assay, and this was probably due to certain attributes of the complex formation that are not known for ornidazole. These attributes outweigh any loss in efficacy in the free radical pathway following complex formation. This is certainly an advantage of complex formation and helps to improve the therapeutic index. This study has attempted to look at some of the possible reasons why the complex performs better than ornidazole. One reason is its ability to bind to DNA better than ornidazole does, and this can be understood by following the interaction of ornidazole and its Zn(ii) complex with calf-thymus DNA using cyclic voltammetry. Therefore, this study showed that despite a decrease in R–NO_2_˙^−^, the complex does not compromise its efficacy, and this was examined using a biological target. In addition, the complex is likely to have less toxic side effects on the host of the disease-causing microbes.

## Abstract 2

A monomeric complex of Zn^II^ with ornidazole [Zn(Onz)_2_Cl_2_] decreases formation of the nitro-radical anion (R–NO_2_˙^−^), and this is realized by recording it in an enzyme assay using xanthine oxidase, which is a model nitro-reductase.

## Graphical abstract

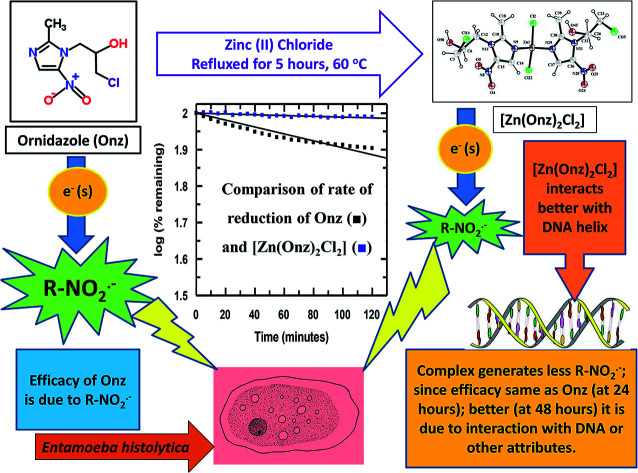

## 1. Introduction

5-Nitroimidazoles are important molecules with established efficacy and have been used to combat pathogenic microbes such as anaerobic bacterial and parasitic infections.^1–4^ They are reductively activated in hypoxic cells, after which they undergo redox recycling or decompose to form products that are cytotoxic.^1–4^ Over time, as more and more compounds in this family have been used, it was revealed that adverse drug reactions, neurotoxic side effects and drug resistance were some of the challenges associated with these drugs that require attention.^1–4^ 5-Nitroimidazoles are also potential radiosensitizers used in radiotherapy for cancers.^5–8^ Metronidazole, tinidazole and ornidazole (Onz) are the three most important molecules of this family that have made their way to the clinics and are being used in a number of pharmaceutical preparations for different reasons.^1–8^ Their efficacy is attributed to the generation of the nitro-radical anion (R–NO_2_˙^−^).^1–10^ In order to tackle infections caused by parasites, these molecules are first reduced by the enzyme pyruvate ferredoxin oxidoreductase (PFOR) that acts as an electron sink.^9,10^ Reduction of the nitro group prepares them for entry into cells by passive diffusion, creating a favorable concentration gradient.^9,10^ After entering the target cells, the anti-microbial toxicity of 5-nitroimidazoles depends on the reduction of the nitro moiety to R–NO_2_˙^−^ and other active species such as nitroso and hydroxylamine derivatives. R–NO_2_˙^−^ binds to DNA, disrupting or breaking strands, leading to cell death.^9,10^ As radiosensitizers, they interact with the radicals formed on DNA following the interaction of the latter with the products of the radiolysis of water, forming R–NO_2_˙^−^, which thereafter enhances strand unwinding or strand breaks.^6–8^

Unfortunately, the same R–NO_2_˙^−^ ion is associated with the neurotoxic side effects, particularly when there is prolonged use of such molecules.^9–11^ In such a situation, the aspects of neurotoxicity, or other forms of side effects, become a matter of concern, creating a need to control the generation of R–NO_2_˙^−^. Too much generation of these reactive intermediates for this class of drugs and others can often cause more harm than good.^12–15^ Hence, generating the correct amount or making it available through slow chemical release is becoming an important aspect of research.^10–15^ This study reports the regulation of R–NO_2_˙^−^ for a specific cause, and this is achieved through the complex formation of one of the members of the 5-nitroimidazole family (ornidazole) with Zn^II^, which can likely generate the correct amount necessary for cytotoxicity of a biological target (axenic *Entamoeba histolytica*). In addition, what the drug compromises for by forming less free radical species, (R–NO_2_˙^−^), it makes up for by other attributes of complex formation. Previously, a Zn^II^ complex of metronidazole was also shown to be very active as an anticancer agent on a number of cancer cell lines.^16^ Hence, such complexes of the 5-nitroimidazole family containing a relatively non-toxic metal ion (Zn^II^) might be useful cytotoxic agents against a number of diseases.

## 2. Experimental section

### 2.1 Materials and methods

Ornidazole [purity (HPLC): >98.0%; melting point: 90.0 to 94.0 °C] was purchased from TCI, Japan. Zinc(ii) chloride (ZnCl_2_, assay (complexometric) 98.0–100.0%, melting point: 293 °C) was purchased from E. Merck, India. Xanthine oxidase (XOD) isolated from cows' milk was obtained as a suspension in ammonium sulphate solution from Sigma Aldrich. Hypoxanthine and calf-thymus DNA were purchased from Sisco Research Laboratories, India. Calf-thymus DNA was dissolved in triple-distilled water using proper electrolytes. Its concentration was determined in terms of nucleotides, taking *ε*_260_ = 6600 mol^−1^ dm^3^ cm^−1^. Tris buffer solution (Spectrochem Pvt. Ltd., India) and NaCl (AR, Merck, Germany) were used to maintain physiological conditions.

#### 2.1.1 Synthesis of a monomeric complex of ornidazole with Zn(ii)

A solution of ornidazole (0.8785 g in 25 mL, 4 mmol) in methanol was added to a solution of ZnCl_2_ (0.2725 g in 25 mL, 2 mmol) in methanol. The mixture was warmed under reflux to a temperature of 60 °C for 6 hours. After a week, a white crystalline compound was obtained by very slow evaporation of the solvent. The solvent was filtered and the solid mass was collected. The filtered product was re-crystalized using a 1 : 1 aqueous-methanol mixture. This was done three times. A pure complex was obtained. Elemental analysis was performed on a PerkinElmer 2400 Series-II CHN analyzer. Analysis: calc. (%) for C_14_H_20_Cl_4_N_6_O_6_Zn, C: 29.21; H: 3.50; N: 14.61. Found: C: 29.12; H: 3.26; N: 15.22.

#### 2.1.2 Solution of the structure of the complex by refinement from X-ray powder diffraction data

Powder X-ray diffraction (PXRD) data were collected at ambient temperature (25 °C) on a Bruker D8 Advance diffractometer operating in reflection mode with Cu Kα_1_ radiation of wavelength 1.540562 Å. The generator was set at 40 kV and 40 mA. The data was collected in the 2*θ* range of 4–60° with a 0.02° step size and a 5 s per step.

Indexing and Pawley refinement of the PXRD pattern of the complex was carried out using the Reflex module of Material Studio.^17^ The PXRD pattern was indexed by the TREOR 90 program^18^ for the first 20 peaks. Indexing revealed that the complex crystallizes in an orthorhombic system with *a* = 10.411(2) Å, *b* = 7.759(2) Å and *c* = 27.730(1) Å. Pawley refinements^19^ were performed in the 2*θ* range of 5–60° on the unit cell. Peak profiles, zero-shift, background and unit-cell parameters were refined simultaneously. Peak profiles were refined by the pseudo-Voigt function with Berar–Baldinozzi asymmetry correction parameters. The background was refined using a 20th-order polynomial. Refinement yields of *R*_p_ = 6.38% and *R*_wp_ = 4.52% were determined. Statistical analysis gave *Pna*2_1_ (33) as the most likely space group.
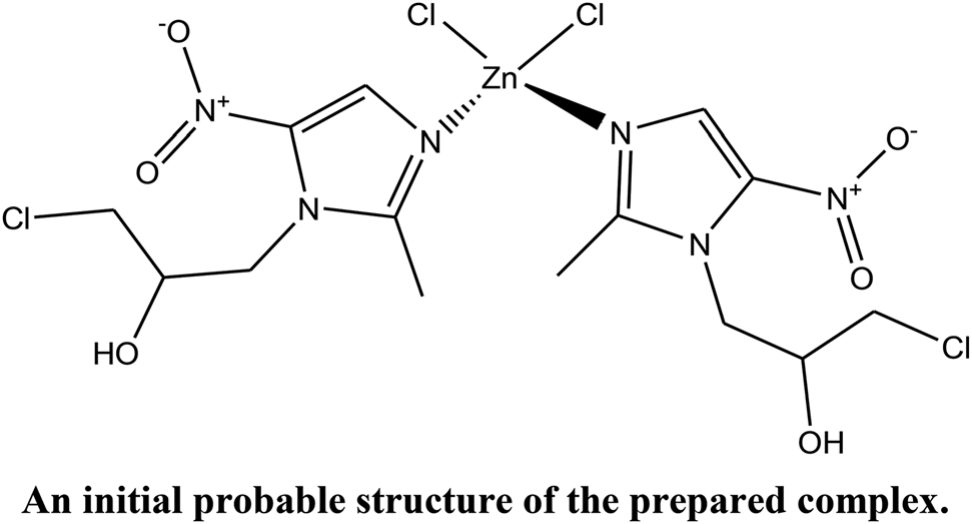


Firstly, an initial molecular structure was drawn using ACD/ChemSketch (shown above) and its geometry was optimized with the help of MOPAC2016 to obtain a reference structural model for the reflex powder solve module. This initial structure (model), consisting of two Onz complexes as ligands, one Zn^II^ ion and two Cl^−^ ions, was then imported into the new cell. After assigning motion groups to different fragments, the structure was solved to represent an approximate structure of the complex.

**Fig. 1:**
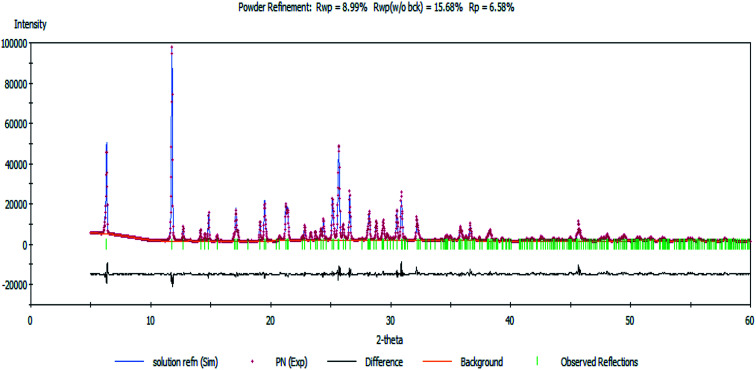
The Rietveld refinement plot showing an agreement between the calculated and measured X-ray powder diffractograms of the complex.

Next, a Rietveld refinement was carried out in order to obtain the final structure using the Reflex Powder Refinement module of Material Studio. To improve the agreement between the calculated and experimental powder diffraction patterns, different parameters such as pseudo-Voigt profile parameters, background parameters, cell constants, zero point of diffraction pattern, position and orientation of motion groups, dihedral angles within the Onz moieties, Berar–Baldinozzi asymmetry correction parameters and March–Dollase preferred orientation correction parameters were optimized step-by-step until the best agreement between the calculated and experimental powder diffraction patterns emerged (Fig. 1).

Thermal parameters were set as the global isotropic atom displacement parameters and were refined thereafter. The final *R*-factors of *R*_p_ = 6.58% and *R*_wp_ = 8.99% were obtained using the following unit cell parameters: space group = *Pna*2_1_, *a* = 10.407(7) Å, *b* = 7.756(5) Å, *c* = 27.725(5) Å and *V* = 2238.20(5) Å^3^. The crystal data is summarized in Table 1. The CSD number of our inorganic structure is 1987809.

**Table:** Summary of the crystal data structural refinement results of the complex^a^

Formula	C_14_H_20_Cl_4_N_6_O_6_Zn
Molecular weight (g)	575.55
Absorption coefficient (*μ*) (mm^−1^)	1.619
Crystal system	Orthorhombic
Space group, *Z*	*Pna*2_1_, 4
*a* (Å)	10.407(7)
*b* (Å)	7.756(5)
*c* (Å)	27.725(5)
*V* (Å^3^)	2238.20(5)
*T* (K)	298
Wavelength (Å)	1.540562
*θ* range used for refinement (°)	5–60
Reliability factors	*R*_p_ = 0.0658
	*R*_wp_ = 0.0899

a *R*_p_ = Σ|*cY*^sim^(2*θ*_i_) − *I*^exp^(2*θ*_i_) + *Y*^back^(2*θ*_i_)|/Σ|*I*^exp^(2*θ*_i_)|. *R*_wp_ = {*w*_p_[*cY*^sim^(2*θ*_i_) − *I*^exp^(2*θ*_i_) + *Y*^back^(2*θ*_i_)]^2^/Σ*w*_p_[*I*^exp^(2*θ*_i_)]^2^}^1/2^, and *w*_p_ = 1/*I*^exp^(2*θ*_i_). *R*_1_ = ∑‖*F*_o_| − |*F*_c_‖/∑|*F*_o_|. w*R*_2_ = [∑*w*(*F*_o_^2^ − *F*_c_^2^)^2^/∑*w*(*F*_o_^2^)^2^]^1/2^.

### 2.2 Physical measurements

The UV-vis spectrum of Zn(Onz)_2_Cl_2_ was recorded on a JASCO V-630 spectrophotometer, JASCO, Japan. FTIR of the solid sample on a KBr pellet was recorded on a PerkinElmer RX-I spectrophotometer. Elemental analysis of the complex was carried out on a PerkinElmer 2400 Series-II CHN analyzer.

### 2.3 Enzyme assay

The method used xanthine oxidase (XOD) as a model nitro-reductase.^10,11,20^ Hypoxanthine was the reducing substrate, while Onz and the Zn^II^ complex were electron acceptors. 225 μL of an XOD suspension was diluted to 1.5 mL with 0.025 M phosphate buffer (pH 7.4) in a quartz cuvette that was sealed with a rubber septum. Oxygen was purged out by passing argon gas through the solution. The enzyme (XOD), with a specific activity of 0.3 units per mg of protein, contained ∼10 units in 1.5 mL. In another quartz cuvette, 1.0 mL hypoxanthine (0.01 M) in 0.1 M phosphate buffer (∼pH 7.4) was taken, along with 125 μL of 1600 μM Onz, and the complex was dissolved in DMF. The volume was made up to 2.0 mL with the help of phosphate buffer (0.1 M). The cuvette was sealed with a rubber septum and oxygen was purged out by passing argon gas through the solution. To initiate the reaction, 500 μL of deoxygenated enzyme solution that was kept in another cuvette was added with the help of a gas-tight syringe to the degassed solution containing hypoxanthine and the test compounds. The final assay solution (2.5 mL) had 0.2 units per mL of XOD, 80 μM of Onz or its complex and 4 mM of hypoxanthine. The cuvette was inverted to mix and was monitored using UV-vis spectroscopy against a buffer-DMF blank. A spectrum of the solution was taken every 5 minutes for 2 hours during the assay. A change in absorbance at 320 nm was noted for Onz and for the complex.

### 2.4 Cyclic voltammetry

Cyclic voltammetry (CV) experiments were carried out on a Metrohm Autolab electrochemical analyzer. A conventional three-electrode system was used, consisting of glassy carbon as the working electrode, a platinum wire as the counter electrode and Ag/AgCl in satd KCl as the reference electrode. Reduction of the nitro group in Onz and its Zn^II^ complex was followed.^21–26^ Before performing cyclic voltammetry on an experimental solution, it was very carefully degassed for 30 min using high-purity Ar. The results were analyzed according to the Randles–Sevcik equation [eqn (1)].^27,28^

1 *i*_pc_ = (2.69 × 10^5^)*n*^3/2^*D*_0_^1/2^*ACν*^1/2^

*i*_pc_ refers to the current in amperes at the cathodic peak potential, *n* denotes the total number of electrons involved in the electrochemical reduction, *D*_0_ is the diffusion coefficient of the species, *A* denotes the area of the electrode in cm^2^, *C* is the concentration of the substance in moles cm^−3^ and *ν* is the scan rate in V s^−1^.

### 2.5 DNA binding

Although the complex has absorption at 320 nm, its interaction with DNA was not tracked at that wavelength since DNA has a *λ*_max_ at 260 nm, and the tail of its absorbance peak extends up to 310 nm, which would therefore interfere with the absorbance of the complex. This, in turn, could affect the correct determination of the change in absorbance, upon which a titration of the complex with calf-thymus DNA, leading to the evaluation of a binding constant, is based.^10,11,29^ Hence, cyclic voltammetry was used to study DNA interaction following a reduction of the nitro group of Onz that is present as a ligand in the complex.^10,11,29–32^ A 30 mL solution containing the complex (100 μM) was used. Calf-thymus DNA was gradually added to the solution and cyclic voltammetry was performed. 20 mM Tris buffer and 120 mM NaCl were used to maintain the pH and ionic strength of the medium, respectively, during the titration. The change in current (Δ*I*) served as a measure of the extent to which the complex interacts with the calf-thymus DNA. The change in current (Δ*I*) was subsequently used in standard equations (ESI†), yielding values for the binding constant and the site size of interaction.^10,11,29–32^ Glassy carbon was used as the working electrode while a platinum wire and Ag/AgCl, satd KCl were used as the counter and reference electrodes, respectively. The experimental solution was degassed for 15 minutes after every addition of DNA, using high-purity Ar. Voltammograms were recorded at a scan rate of 100 mV s^−1^.

In the medium used, Zn(Onz)_2_Cl_2_ undergoes reduction at −945 mV. As the concentration of the calf-thymus DNA was gradually increased, the peak current due to the complex gradually decreased. Based on the change in peak current (Δ*I*), binding constant values were evaluated considering the equilibrium in eqn (2) where *K*_d_ denotes the dissociation constant related to the process.2
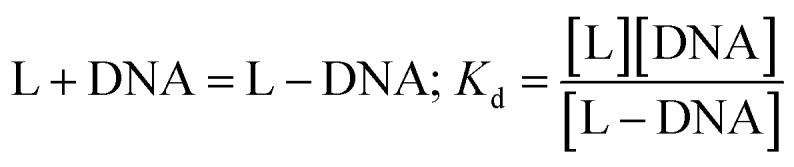


For the complex, [Zn(Onz)_2_Cl_2_], a decrease in peak current during the titration is a consequence of structural changes following the binding of the complex to DNA that gradually disables nitro groups from showing a response in cyclic voltammetry results, and this was therefore followed for the evaluation of the binding constant.^10,11,29–32^

### 2.6 Biological assay on an amoeba strain

Axenic *Entamoeba histolytica* strain HM1:IMSS was maintained and grown in TYIS-33 medium supplemented with 15% adult bovine serum, 1× diamond vitamin mix and antibiotics (0.3 units per mL penicillin and 0.25 mg mL^−1^ streptomycin) at 35.5 °C. The cells were sub-cultured twice a week.^33^*Entamoeba* cells were taken from the log phase and an *in vitro* drug sensitivity assay was carried out for 24 hours and 48 hours, respectively.^34^

An *in vitro* sensitivity assay was carried out on a 96 micro-titre plate following a protocol described previously.^34^ Briefly, stock solutions (10 mM) of different compounds were prepared in DMSO and further diluted with their respective media to obtain the desired concentration. These were added to the well of a micro-titre plate, in triplicate. Strain HM1:IMSS was harvested at the log phase and pelleted at 600 g for 5 min and then a cell suspension was made. Cells were counted using a haemocytometer. Equal volumes of the cell suspension of the axenic strain HM1:IMSS were added to wells containing different compounds so that the total number of trophozoites per well was 3 × 10^5^. Final compound concentrations in the rows down the plate were 200, 100, 50, 25, 12.5, 6.25, and 3.125 μM, respectively. Appropriate controls in triplicate were included in each plate with DMSO, ZnCl_2_ and non-treated media (allowing for 100% growth). Subsequently, the micro-titre plate was placed in an incubation bag with an aerocult mini sachet to maintain an anaerobic atmosphere. The incubation bag was sealed and placed in an incubator where the temperature was 35 °C.

Cell growth was monitored on a daily basis at 24 hours and 48 hours by comparing the compound contained in the wells with the controls in same rows using an inverted microscope. Plates were properly examined and each well was scored according to its well coverage, cell mobility, and cell rounding. + was given to 30% well coverage area with rounded cells and ++++ for fully covered wells with pseudopodal movement. MIC was considered as the lowest concentration of a compound where a score of + could be given.

An *in vitro* compound susceptibility (Trypan blue) assay was carried out where 3 × 10^5^ trophozoite was added in 3 replicate wells containing previously diluted different concentrations of compounds. A non-treated control (100% growth) was included in each plate. Culture plates were sealed and incubated for 24 hours and 48 hours, respectively, at 37 °C. Trophozoite growth was determined after 24 hours and 48 hours by counting with a microscope using a 0.4% trypan blue assay.^35^

## 3. Results and discussions

### 3.1 Characterization of the complex

#### 3.1.1 Crystal structure from powder X-ray diffraction data

**Fig. 2:**
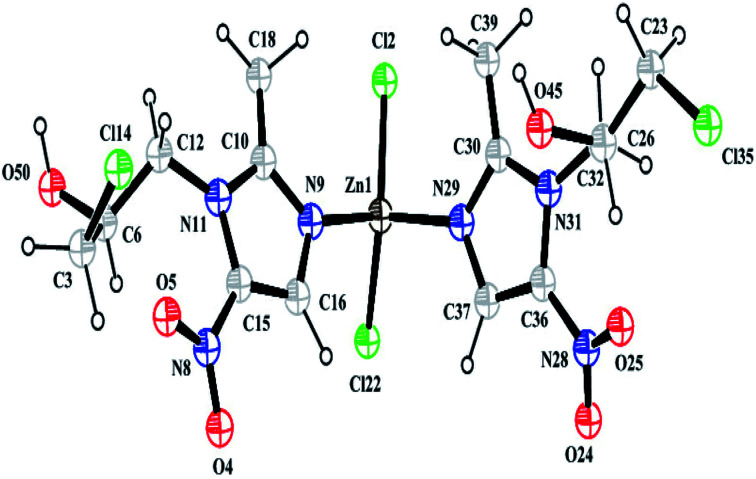
A perspective view of the complex.

Structural analysis revealed that the complex crystallizes in an achiral *Pna*2_1_ space group belonging to the orthorhombic system and that it has cell dimensions of *a* = 10.407(7) Å, *b* = 7.756(5) Å, and *c* = 27.725(5) Å. The asymmetric unit of the complex consists of one Zn^2+^ ion, two Onz moieties and two Cl^−^ ions. An ORTEP diagram is shown in Fig. 2. The metal center exhibits a four-coordinated slightly distorted tetrahedral geometry. The two N atoms (N9 and N29) of two different Onz moieties and two Cl^−^ ions (Cl2 and Cl22) occupy the four corners of a tetrahedron surrounding the Zn^2+^ ion. Zn–N bond distances were ∼2.017 Å, while Zn–Cl bond distances were ∼2.239 Å. Two imidazole nitrogen atoms of two different Onz moieties bind to the metal center in a syn–syn fashion. Some of the coordinated bond distances and bond angles are listed in Table 2.

Some selected bond lengths (Å) and bond angles (°) of the complex

**Table:** 

Zn1–Cl2	2.238(7)	Zn1–Cl22	2.238(5)	Zn1–N9	2.017(2)
Zn1–N29	2.017(3)	Cl14–C3	1.779(7)	Cl35–C23	1.779(9)
C26–C32	1.549(3)	C3–C6	1.533(9)	C6–C12	1.549(4)
C10–C18	1.470(7)	C15–C16	1.408(9)	C23–C26	1.533(9)
O45–C26	1.417(4)	O50–C6	1.417(5)	N31–C32	1.470(6)

**Table:** 

Cl2–Zn1–Cl22	134.95	O50–C6–C3	108.88
Cl2–Zn1–N9	106.13	O50–C6–C12	111.68
Cl2–Zn1–N29	98.84	C3–C6–C12	110.57
Cl22–Zn1–N9	98.90	Cl22–Zn1–N29	106.16
N9–Zn1–N29	111.32	N11–C12–C6	111.25
Cl35–C23–C26	108.88	O45–C26–C23	108.89
O45–C26–C32	11.68	C23–C26–C32	110.56
N31–C32–C26	111.25	Cl14–C3–C6	108.89

#### 3.1.2 UV-vis spectroscopy of ornidazole and its Zn^II^ complex in different solvents

Ornidazole and Zn(Onz)_2_Cl_2_ were dissolved in different solvents (acetonitrile, DMF, methanol and water) with concentrations of 10^−4^ M. The absorption spectra of Onz and of the complex showed a strong response in the UV region from 260 nm to 320 nm (Fig. S1 and S2 in the ESI,† respectively). Absorption bands were similar (Table 3) and can be assigned to the intra-ligand charge transfer.

**Table:** Absorption of ornidazole and Zn^II^-ornidazole in different solvents

Compound	Acetonitrile		DMF		Methanol		Water	
	*λ*_max_ (nm)	Absorption intensity	*λ*_max_ (nm)	Absorption intensity	*λ*_max_ (nm)	Absorption intensity	*λ*_max_ (nm)	Absorption intensity
Onz	320	0.9335	324	1.0638	312	0.9519	318	1.2287
Zn(Onz)_2_Cl_2_	318	1.7287	324	1.2643	310	1.5615	320	1.1576

#### 3.1.3 Analysis of the IR spectra of ornidazole and its monomeric Zn^II^ complex

The IR spectrum of Onz (Fig. S3 in the ESI†) shows a band at 1538.21 cm^−1^, which can be assigned to the *ν*_C=N_ stretching vibration of the imidazole ring that shifts to a higher wavenumber (1565.80 cm^−1^) in Zn(Onz)_2_Cl_2_ (Fig. S4 in the ESI†), suggesting coordination of Zn^II^ by the imidazole nitrogen. Two NO_2_ stretching vibrations, *ν*_as_ 1482 cm^−1^ and *ν*_s_ 1380 cm^−1^, in the complex were similar to those in Onz, indicating that –NO_2_ does not participate in coordination of the metal center (Table S1 in the ESI†).^36^

#### 3.1.4 Mass spectrometry of the complex

**Fig. 3:**
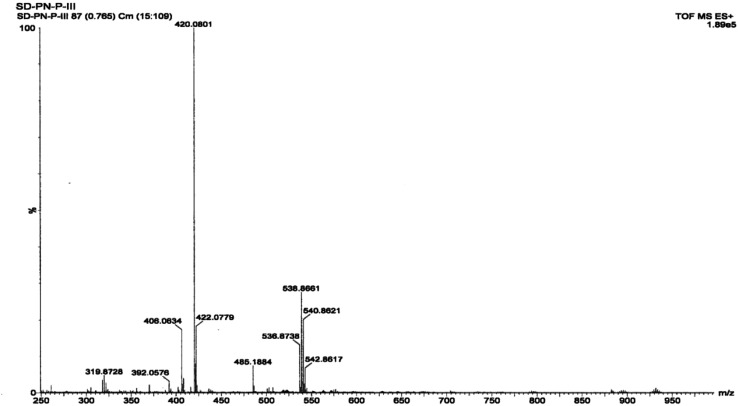
Mass spectrum of the Zn(Onz)_2_Cl_2_ complex.

PXRD data, showing the structure of the complex, suggest that its molecular formula is Zn(Onz)_2_Cl_2_. Hence, a molecular ion peak in the mass spectrum should be found in the range from *m*/*z* = 572 to *m*/*z* = 584 (considering isotope effects due to Cl present in ornidazole, Cl present in the coordination zone around the metal ion and due to isotope effects for Zn; details in the ESI†). However, the molecular ion peak, which was expected in the above mentioned region, was not seen in Fig. 3, although indications that were not very prominent were seen in the region, suggesting that the molecular ion was not very stable to the electrospray stimuli. A cluster of peaks were obtained at *m*/*z* values of 536.8239, 538.8197, 540.8192 and 542.8129 that may be attributed to a species formed from the complex following the loss of a Cl atom coordinated to Zn (likely peaks are shown in the ESI,† based on isotopic distribution). Only those peaks for which the relative abundance of isotopes is high were actually found, and not all possibilities are indicated in the ESI.† The peak at *m*/*z* = 485.1884 can be attributed to the formation of a fragment from the loss of two Cl atoms from the coordination sphere and an –OH group from an ornidazole moiety present as a ligand in the complex (details in the ESI†). Prominent experimental peaks with *m*/*z* values of 420.0368 and 422.0334 were also detected in the mass spectrum and were assigned to a fragment formed from the molecular ion where –CH_3_ and –NO_2_ depart from each Onz moiety, along with the loss of a Cl atom from either of the two Onz moieties in the complex (*m*/*z*_theo_ ranges from 420 to 424; possibilities for such a fragment are shown in the ESI†).

#### 3.1.5 Cyclic voltammetry studies

Cyclic voltammetry of Zn(Onz)_2_Cl_2_ (Fig. S5†) and of Onz (Fig. S6†) were performed; the former in aqueous-methanol and in pure aqueous solution, while the latter was carried out in aqueous solution at a scan rate of 0.1 V s^−1^. The complex showing a peak at −0.925 V against Ag/AgCl, satd KCl [Fig. S5†] was assigned to the reduction of the –NO_2_ moiety in Onz. A cyclic voltammogram of Onz alone (Fig. S6†) showed a peak at −0.827 V. Voltammograms for the reduction of the nitro group in the complex were obtained at other scan rates as well. *i*_pc_ was plotted against the square root of the scan rate using the Randles–Sevcik equation [Fig. S7†]. A straight line passing through the origin suggested that the complex undergoes reduction in a diffusion-controlled pathway and that there is no adsorption on the electrode surface.

In aprotic media, 5-nitroimidazoles undergo reversible one-electron reduction to form the nitro radical anion (R–NO_2_˙^−^). This is then followed by a three-electron reduction to hydroxylamine derivatives. For most 5-nitroimidazoles, the first step is reversible whilst the second step is not reversible, and this can be seen from studies on metronidazole (eqn (3) and (4)).^26^ In aqueous solution, the two steps cannot be carried out separately and a single-step four-electron reduction is observed (eqn (5)).^26,37^

3 R–NO_2_ + e^−^ → R–NO_2_˙^−^

4 RNO_2_˙^−^ + 3e^−^ + 4H^+^ → RNHOH + H_2_O

5 RNO_2_ + 4e^−^ + 4H^+^ → RNHOH + H_2_O

### 3.2 Binding of the complex to DNA

**Fig. 4:**
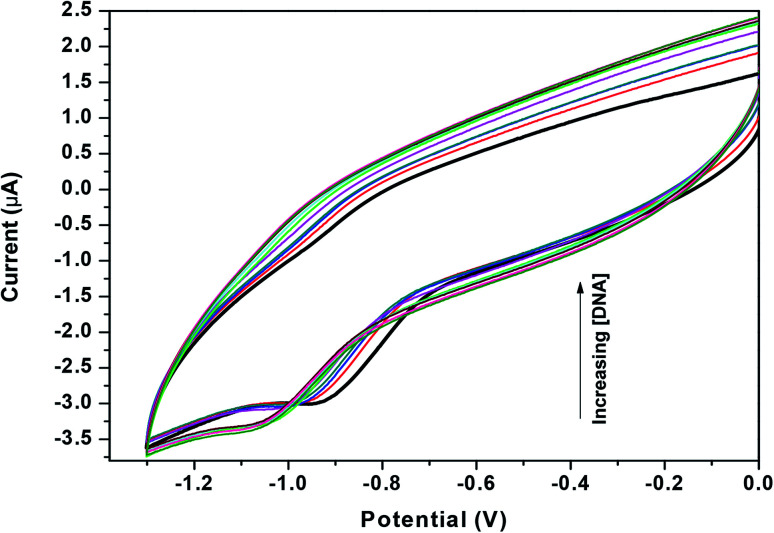
Cyclic voltammograms for Zn(Onz)_2_Cl_2_ in the absence (dark black line) and presence of different concentrations of calf-thymus DNA, [Zn(Onz)_2_Cl_2_] = 100 μM; [NaCl] = 120 mM; pH = 7.4; *T* = 301 K.

Zn(Onz)_2_Cl_2_ was titrated with calf-thymus DNA under physiological conditions using cyclic voltammetry. The reduction peak of the complex, identified at −0.925 V (against Ag/AgCl, satd KCl), was used to follow the titration. Upon addition of the calf-thymus DNA, the cathodic peak current (*i*_pc_) gradually decreased with a shift to a more negative potential (Fig. 4). This shift to a more negative potential indicated that there is an interaction between the complex and the DNA. As more DNA is added, it becomes increasingly difficult for the complex to show a response in cyclic voltammetry (indicated by a shift to more negative potential), as the complex becomes progressively bound to the DNA.^27^

**Fig. 5:**
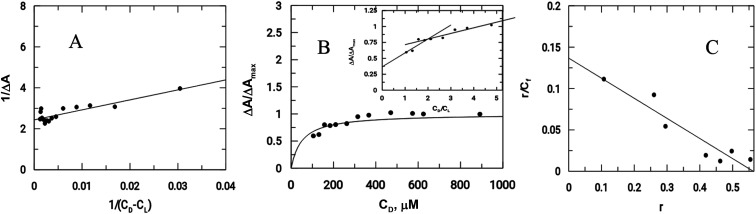
Interaction of calf-thymus DNA with Zn(Onz)_2_Cl_2_. (A) Double reciprocal plot, (B) non-linear plot, (C) Scatchard plot; [Zn(Onz)_2_Cl_2_] = 100 μM; [NaCl] = 120 mM; pH = 7.4; Temp. = 28 °C.

As previously mentioned, binding of the complex to DNA results in the redox active nitro groups on both Onz ligands to gradually lose their ability to show a response in the cyclic voltammetry tests. Therefore, this gradual decrease in peak current could be used as a measure of the extent of interaction between the complex and calf-thymus DNA. Apparent binding constant (*K*_app_) values were evaluated from a linear plot (eqn (S3) in the ESI†) and also from non-linear square fit analysis results (eqn (S5) in the ESI†). The results are shown in Fig. 5 and Table 4.^10–12,29–32^

**Table:** Binding constant values for the interaction of the complex with calf-thymus DNA

Compound	Apparent binding constants *K*_app_ × 10^−4^ (M^−1^)			Site size (*n*_b_) from molar ratio plot	Overall binding constant [*K*′ × 10^−4^ (M^−1^) *K*′ = *K*_app_ × *n*_b_]			Overall binding constant *K*′ × 10^−4^ (M^−1^) from the Scatchard plot	Site size (*n*_b_) from the Scatchard plot
	From the double reciprocal plot	From the non linear-fit	From the double reciprocal plot with intercept = 1		Using *K*_app_ From the double reciprocal plot	Using *K*_app_ from the non linear-fit	Using *K*_app_ from the double reciprocal plot with *y*-intercept = 1		
Onz^10^	1.71	1.46	—	1.5	2.47	2.11	—	2.77	1.39
Zn(Onz)_2_Cl_2_	4.85	2.07	2.33	2.0	9.34	3.98	4.49	23.50	1.85

Another important parameter for binding to DNA is the site size of interaction (*n*_b_). This provides an estimate of the number of nucleotides that are bound to the material during the interaction. This value was 2 for Zn(Onz)_2_Cl_2_ binding to DNA, and this could be calculated from the molar ratio plot (inset of Fig. 5B). Since *K*_app_ provides the binding constant value of a substance binding to an isolated site, an overall binding constant (K*) can be obtained by multiplying *K*_app_ with the site size of interaction (*n*_b_).^38^ These values are provided in Table 4. The binding constant values for Zn(Onz)_2_Cl_2_ were higher than for Onz (determined earlier),^10^ indicating that this is an attribute of complex formation. The site size of interaction (*n*_b_) of Onz (reported earlier)^10^ was in the range of 1.39 to 1.5, while that of the monomeric Zn^II^ complex was found in the range from 1.85 to 2 (from two different methods of analysis), suggesting that the complex with two Onz ligands bound to Zn^II^ might be involved in two types of interaction with the DNA. In one interaction, the complex interacts in the same manner as free Onz does, *i.e.* one of the two Onz ligands bound to Zn^II^ in the complex interacts with the DNA while the other does not. In the other mode, both Onz ligands bound to Zn^II^ interact with the DNA. Hence, the values obtained for the site size of interaction during the experiments were intermediate between the two values obtained. While the inset of Fig. 5B (the molar ratio plot) provides a value of 2.0, Fig. 5C (the modified Scatchard plot) provides a value of 1.85. This may be explained by the obtained values between 1.5 (if one Onz ligand interacts with DNA) and 3.0 (if both Onz ligands interact), based on our earlier findings with Onz itself interacting with calf-thymus DNA.^10^

### 3.3 Enzyme assay

Xanthine oxidase catalyzes the oxidation of hypoxanthine to xanthine and then to uric acid in the presence of oxygen. Under anaerobic conditions, this action does not occur as there is no substrate to accept the electrons. Therefore, under anaerobic conditions (*i.e.* in the absence of oxygen) if an electron acceptor molecule is present in the system, the above oxidation of hypoxanthine to xanthine and to uric acid becomes possible. Nitroimidazoles are good electron acceptors and so can participate in the above reaction, forming the nitro radical anion (RNO_2_˙^−^). Therefore, it can be seen that, using an enzyme assay, the ability of 5-nitroimidazoles and their modified forms to reduce and to know whether the nitro-radical anion generated is stable for enough time to cause a change in absorbance of the compound on which it is formed, can be recorded as a gradual decrease in absorbance. In contrast, if RNO_2_˙^−^ does not form or if its generation is not stable for long, *i.e.* it can revert back to its original form (the nitro group) by any pathway, it can be inferred that there is a decrease in the formation of RNO_2_˙^−^, leading to either almost no decrease in absorbance of the original compound or to a decrease that is considerably less than where reduction leads to a stable RNO_2_˙^−^ ion.

**Fig. 6:**
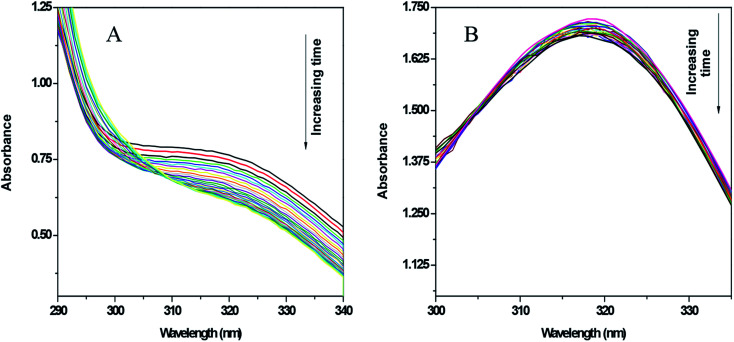
UV-vis spectra of Onz (A) and Zn(Onz)_2_Cl_2_ (B) in the presence of hypoxanthine in 5% DMF and xanthine oxidase. Spectra of each compound were taken at intervals of 5 minutes for 2 hours. Enzyme activity = 0.2 units per mL in XOD and compound concentration = 80 μM.

As mentioned earlier, xanthine oxidase acts as a nitro-reductase that reduces the nitro group, where hypoxanthine is the source of the electron. In a previous study, we reported that the formation of a nitro radical anion by a Cu^II^ complex of ornidazole was significantly less than with Onz alone, and this was recorded by UV-vis spectroscopy.^11^ In this study, we tried to identify the amount of the nitro radical anion formed by the Zn^II^ complex and see how it compares with that formed by Onz. Since the enzyme assay reduces the nitro group, such a reduction causes a change in the absorption spectra. A continuous decrease in the absorption of a compound during the assay indicates that the nitro group is irreversibly reduced, leading to the destruction of the chromophore. Whilst a gradual decrease in absorbance was observed for Onz (Fig. 6A), in the case of the complex there was no significant decrease (Fig. 6B), indicating that the formation of the nitro radical anion on the Onz ligand bound to Zn^II^ is substantially controlled.

**Fig. 7:**
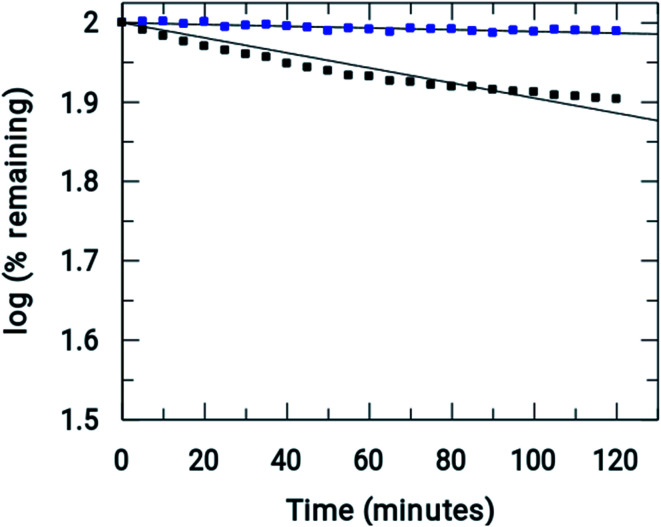
Comparison of the rate of reductions of Onz (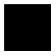) and [Zn(Onz)_2_Cl_2_] (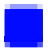) under anaerobic conditions. (initial concentrations = 0.2 U mL^−1^ in XOD, 80 μM in Onz or Zn(Onz)_2_Cl_2_ in the mixture, 4 mM in hypoxanthine and 5% in DMF).

Fig. 7 shows the amount of Onz and of the complex remaining upon completion of the assay. Controlled formation of R–NO_2_˙^−^ following complex formation between Onz and Zn^II^ is important, as it prevents too much generation of R–NO_2_˙^−^. Whilst, on the one hand, this is beneficial as it should make the complex less neurotoxic even during prolonged use, on the other hand, there is a cause for concern because the formation of R–NO_2_˙^−^ is extremely important for drug action of the 5-nitroimidazole family and its decrease might adversely affect its efficacy by not being able to act on disease-causing microbes with the efficiency that is known for Onz. This could allow pathogenic microbes to multiply much faster than at the rate at which they would be killed by this modified version of Onz, *i.e.* Zn(Onz)_2_Cl_2_. Therefore, it is essential to determine the extent to which cytotoxicity is affected following complex formation of Onz with Zn^II^. For this reason, the performances of Zn(Onz)_2_Cl_2_ and Onz were tested on *Entamoeba histolytica*.

### 3.4 Antiparasitic activity on *Entamoeba histolytica* (HM1:IMS Strain)

The inhibition of cell viability of *Entamoeba histolytica* was recorded by the trypan blue assay using different concentrations of Onz and Zn(Onz)_2_Cl_2_ for 24 hours and 48 hours.^35^ MIC values recorded after 24 hours for Onz and Zn(Onz)_2_Cl_2_ on *Entamoeba histolytica* were similar (12.5 μM, Table 5). Upon increasing the exposure time for each compound from 24 hours to 48 hours, the complex was found to be more cytotoxic with an MIC value of 6.25 μM, while for Onz it remained at 12.5 μM. Therefore, this study clearly shows that increasing the exposure time will have a positive effect for the complex, and this may be considered an advantage of complex formation. More importantly, despite a decrease in the formation of R–NO_2_˙^−^ following complex formation, the efficacy was not affected as was previously thought to be the case (speculating that the mechanism of action might suffer in the free radical pathway). Even if we do not consider the data recorded at 48 hours, which is clearly beneficial, and just compare the data recorded at 24 hours, we see the complex is better than Onz, as it has a similar MIC value but added benefits with regard to the toxic side effects, since it generates less R–NO_2_˙^−^. The complex therefore makes up for any compromise it makes in the free radical pathway by other attributes of complex formation, one of which could be its strong binding affinity for DNA, leading to cell death in disease-causing microbes. The other advantage of complex formation, enabling it to perform much better at longer exposure times (48 hours), could be its effective cellular uptake due to the presence of a metal ion in the complex.^39^

**Table:** MIC values for ornidazole and Zn(Onz)_2_Cl_2_ when applied to *Entamoeba histolytica* (HM1:IMS Strain)

Compound	MIC after 24 h	MIC after 48 h
Ornidazole	12.5 μM	12.5 μM
Zn(Onz)_2_Cl_2_	12.5 μM	6.25 μM
ZnCl_2_	>100 μM	>100 μM
DMSO	>100 μM	>100 μM

In an earlier study, we showed that a Cu^II^ complex of an azo-based ligand performed much better on a particular cell line than the ligand itself, and that this was essentially due to effective cellular uptake of the complex (in that case all attributes required for good performance were in fact similar for the complex and the azo compound forming the complex), suggesting that effective cellular uptake is an essential criteria and can make a difference in performance.^39^ Likewise, in this study of Zn(Onz)_2_Cl_2_, the fact that it performs better at longer exposure times (48 hours) could mean that it enters cells of disease-causing microbes much better than Onz alone. The study also showed that both Onz and its Zn^II^ complex diminished the viability *of Entamoeba histolytica* in a concentration dependent manner.

## 4. Conclusions

Despite a significant decrease in the formation of the nitro radical anion in Zn(Onz)_2_Cl_2_, this study showed that there was comparable biological activity of Onz and the monomeric Zn^II^ complex on *Entamoeba histolytica* (HM1:IMS Strain) when incubated for 24 hours. After 48 hours of incubation, the complex performed better than Onz. This indicates that either the nitro radical anion generated by the complex is sufficient for cytotoxic activity on the chosen biological target or, if a compromise is made by the complex in the free radical pathway following a decrease in nitro-radical anion formation, that the cytotoxic activity is unaffected, implying that the complex makes up for the loss in efficacy in the free radical pathway by other attributes of complex formation. Consequently, association of Zn^II^ with Onz is useful as it not only maintains the efficacy of Onz but, by decreasing R–NO_2_˙^−^, it also helps to overcome the associated neurotoxic side effects of Onz. The study also suggests avoiding too much generation of R–NO_2_˙^−^, since the amount is probably in excess of what is required for cytotoxicity and, if left unattended in the system, it results in toxic side effects. Therefore, if a complex like Zn(Onz)_2_Cl_2_ is used instead of Onz, there will be no compromise in cytotoxic activity, but there could be a significant decrease in toxic side effects associated with Onz. This could be a tremendous benefit in the use of 5-nitroimidazoles and their derivatives.

## Conflicts of interest

There are no conflicts to declare.

## Abbreviations

Onz or R–NO_2_: Ornidazole
CV: Cyclic voltammetry
MIC: Minimum inhibitory concentration
R–NO_2_˙^−^: Denotes the nitro radical anion formed either on an ornidazole moiety or on an ornidazole bound to Zn(ii) in the complex

## Supplementary Material

RA-010-D0RA02597F-s001

RA-010-D0RA02597F-s002
