# Supplementary material for: A ZnII complex of ornidazole with decreased nitro radical anions that is still highly active on Entamoeba histolytica
Source: RSC Adv. 2020 Jun 17;10(39):23286–96. doi: 10.1039/d0ra02597f (PMC9054926; doi:10.1039/d0ra02597f)
Supplement: RA-010-D0RA02597F-s001 [file RA-010-D0RA02597F-s001.pdf]

Electronic Supplementary Material (ESI) for RSC Advances.
This journal is © The Royal Society of Chemistry 2020

Supplementary Information to the manuscript

A Zn^{II} complex of Ornidazole with decreased nitro radical anion is still very active on *Entamoeba histolytica*

[a]Promita Nandy, [b]Soumen Singha [c]Neha Banyal, [c]Kasturi Mukhopadhyay, [b]Sanjay Kumar and [a]Saurabh Das*

[a]Department of Chemistry, Inorganic Section, Jadavpur University, Kolkata – 700 032, India.
[a]Department of Physics, Inorganic Section, Jadavpur University, Kolkata – 700 032, India.
[b]School of Environmental Sciences, Jawaharlal Nehru University, New Delhi – 110067, India.
E-mail: dasrsv@yahoo.in

Equations for evaluating binding constant for the interaction of $Zn(Onz)_2Cl_2$ with calf thymus DNA

$$\frac{1}{\Delta I} = \frac{1}{\Delta I_{max}} + \frac{K_d}{\Delta I_{max}(C_D - C_0)} \qquad (SE\ 1)$$

$$K_d = \frac{\left[C_0 - \left(\frac{\Delta I}{\Delta I_{max}}\right)C_0\right]\left[C_D - \left(\frac{\Delta I}{\Delta I_{max}}\right)C_0\right]}{\left(\frac{\Delta I}{\Delta I_{max}}\right)C_0} \qquad (SE\ 2)$$

$$C_0\left(\frac{\Delta I}{\Delta I_{max}}\right)^2 - (C_0 + C_D + K_d)\left(\frac{\Delta I}{\Delta I_{max}}\right) + C_D = 0 \qquad (SE\ 3)$$

$$\frac{r}{C_f} = K(n - r) \qquad \text{(SE 4)}$$

UV-Vis spectra of Ornidazole and Zn^{II}-Ornidazole in different solvents

Fig. S1

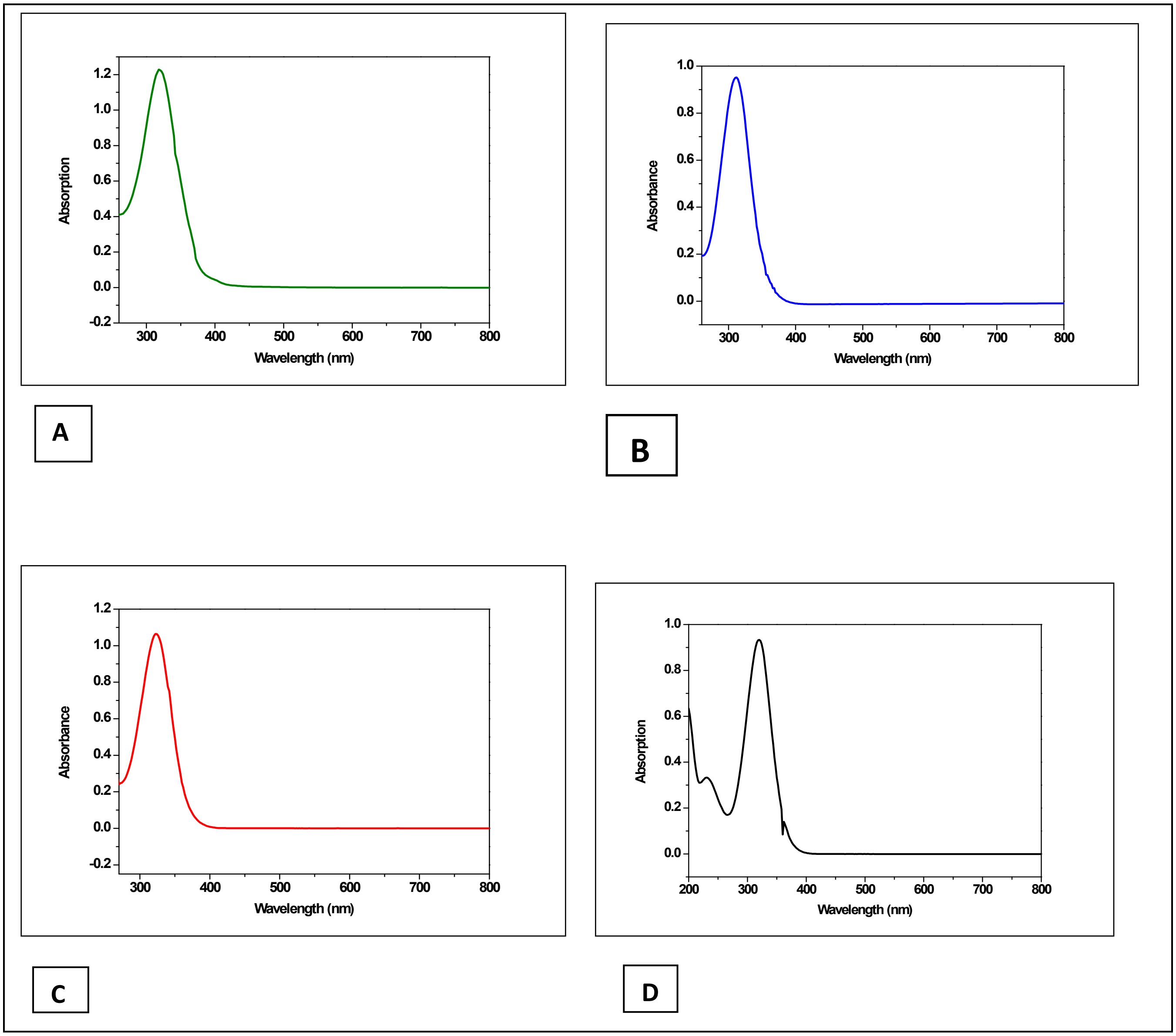

Fig. S1: UV-Vis spectra of Onz in A) water, B) methanol, C) DMF and D) acetonitrile

Fig. S2

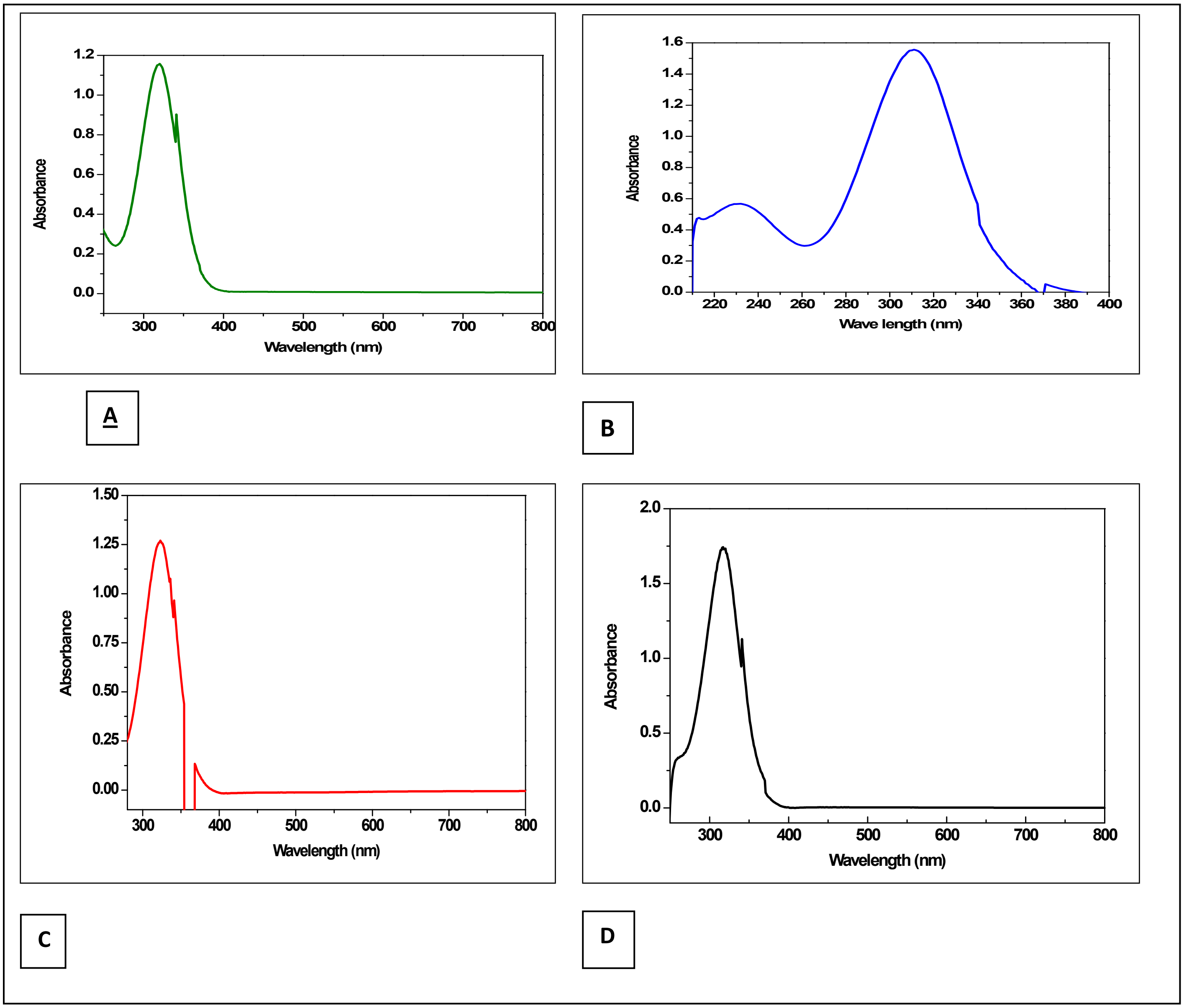

Fig. S2: UV-Vis spectra of $Zn(Onz)_2Cl_2$ in A) water, B) methanol, C) DMF, D) acetonitrile

Table S1: IR stretching frequencies of Ornidazole and $Zn(Onz)_2Cl_2$

Functional group	IR bands (values in cm^{-1})	
	Onz	$[Zn(Onz)_2Cl_2]$
C=N	1538.21	1565.80
NO_2 (ν_s)	1385.92	1380.48
NO_2 (ν_{as})	1471.60	1482.00

Fig. S3

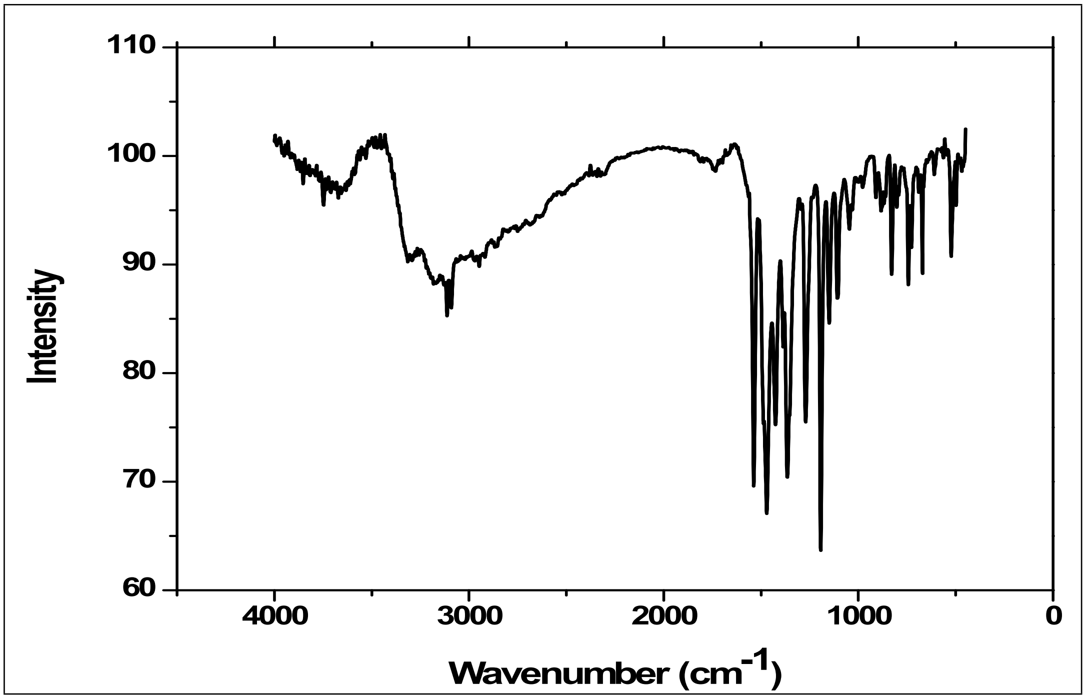

Fig. S3: IR spectrum of Onz

Fig. S4

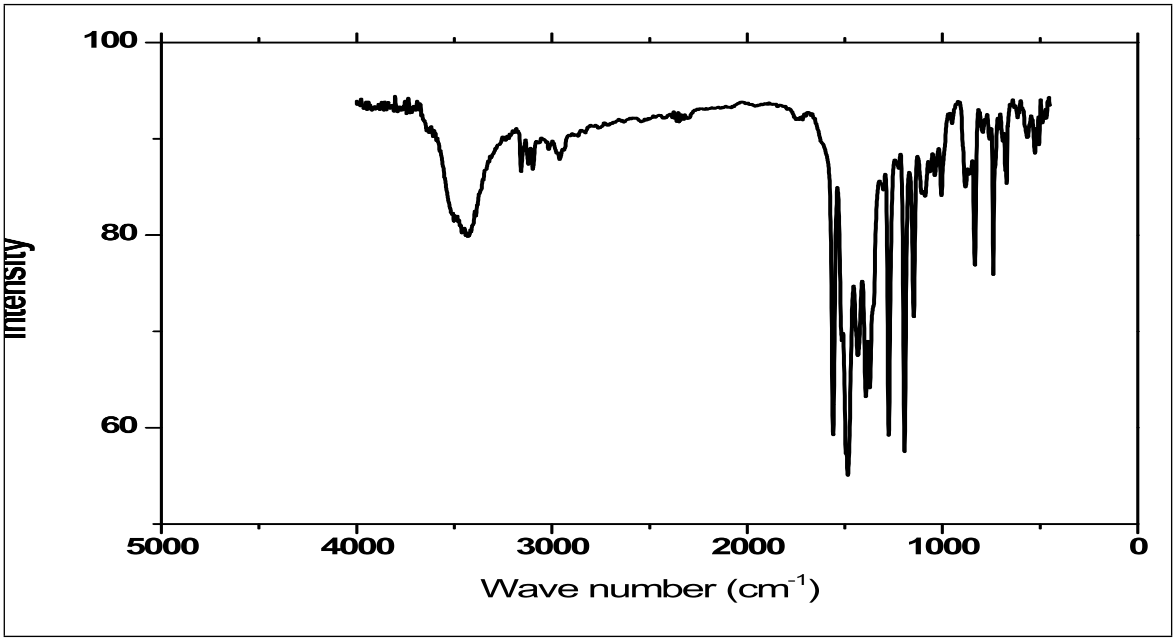

Fig. S4: IR spectrum of $[Zn(Onz)_2Cl_2]$

Related to the mass spectrum of the complex ($C_{14}H_{20}Cl_4ZnN_6O_6$)

Expected molecular ion peaks considering isotope distribution due to Zn [isotopes ^{64}Zn (49.2%), ^{66}Zn (27.7%), ^{67}Zn (4.0%) & ^{68}Zn (18.5%)] and four Cl atoms (isotopes ^{35}Cl & ^{37}Cl).

571.9483: $C_{14}H_{20}{}^{35}Cl_4N_6O_6{}^{64}Zn$;

573.9454: $C_{14}H_{20}{}^{35}Cl_3{}^{37}ClN_6O_6{}^{64}Zn$;

575.9425: $C_{14}H_{20}{}^{35}Cl_2{}^{37}Cl_2N_6O_6{}^{64}Zn$

577.9396: $C_{14}H_{20}{}^{35}Cl{}^{37}Cl_3N_6O_6{}^{64}Zn$

579.9367: $C_{14}H_{20}{}^{37}Cl_4N_6O_6{}^{64}Zn$;

573.9452: $C_{14}H_{20}{}^{35}Cl_4N_6O_6{}^{66}Zn$;

575.9423: $C_{14}H_{20}{}^{35}Cl_3{}^{37}ClN_6O_6{}^{66}Zn$;

577.9394: $C_{14}H_{20}{}^{35}Cl_2{}^{37}Cl_2N_6O_6{}^{66}Zn$

579.9365: $C_{14}H_{20}{}^{35}Cl{}^{37}Cl_3N_6O_6{}^{66}Zn$

581.9336: $C_{14}H_{20}{}^{37}Cl_4N_6O_6{}^{66}Zn$;

574.9463: $C_{14}H_{20}{}^{35}Cl_4N_6O_6{}^{67}Zn$;

576.9434: $C_{14}H_{20}{}^{35}Cl_3{}^{37}ClN_6O_6{}^{67}Zn$;

578.9405: $C_{14}H_{20}{}^{35}Cl_2{}^{37}Cl_2N_6O_6{}^{67}Zn$

580.9376: $C_{14}H_{20}{}^{35}Cl{}^{37}Cl_3N_6O_6{}^{67}Zn$

582.9347: $C_{14}H_{20}{}^{37}Cl_4N_6O_6{}^{67}Zn$

575.9440: $C_{14}H_{20}{}^{35}Cl_4N_6O_6{}^{68}Zn$;

577.9411: $C_{14}H_{20}{}^{35}Cl_3{}^{37}ClN_6O_6{}^{68}Zn$;

579.9382: $C_{14}H_{20}{}^{35}Cl_2{}^{37}Cl_2N_6O_6{}^{68}Zn$

581.9353: $C_{14}H_{20}{}^{35}Cl{}^{37}Cl_3N_6O_6{}^{68}Zn$

583.9324: $C_{14}H_{20}{}^{37}Cl_4N_6O_6{}^{68}Zn$

Explanation for peaks 536.8239, 538.8197, 540.8192 and 542.8129 in the mass spectrum

Possible masses for a fragment formed from the molecular ion due to loss of Cl coordinated to Zn, considering isotope distribution due to Zn [^{64}Zn (49.2%), ^{66}Zn (27.7%), ^{67}Zn (4.0%) & ^{68}Zn (18.5%)] and the three Cl atoms [^{35}Cl (75%) & ^{37}Cl (25%)].

536.9795: $C_{14}H_{20}{}^{35}Cl_3N_6O_6{}^{64}Zn$;

538.9766: $C_{14}H_{20}{}^{35}Cl_2{}^{37}ClN_6O_6{}^{64}Zn$;

540.9737: $C_{14}H_{20}{}^{35}Cl^{37}Cl_2N_6O_6{}^{64}Zn$

542.9708: $C_{14}H_{20}{}^{37}Cl_3N_6O_6{}^{64}Zn$

538.9764: $C_{14}H_{20}{}^{35}Cl_3N_6O_6{}^{66}Zn$;

540.9735: $C_{14}H_{20}{}^{35}Cl_2{}^{37}ClN_6O_6{}^{66}Zn$;

542.9706: $C_{14}H_{20}{}^{35}Cl^{37}Cl_2N_6O_6{}^{66}Zn$

544.9677: $C_{14}H_{20}{}^{37}Cl_3N_6O_6{}^{66}Zn$

539.9795: $C_{14}H_{20}{}^{35}Cl_3N_6O_6{}^{67}Zn$;

541.9746: $C_{14}H_{20}{}^{35}Cl_2{}^{37}ClN_6O_6{}^{67}Zn$;

543.9717: $C_{14}H_{20}{}^{35}Cl^{37}Cl_2N_6O_6{}^{67}Zn$

545.9688: $C_{14}H_{20}{}^{37}Cl_3N_6O_6{}^{67}Zn$

540.9752: $C_{14}H_{20}{}^{35}Cl_3N_6O_6{}^{66}Zn$;

542.9723: $C_{14}H_{20}{}^{35}Cl_2{}^{37}ClN_6O_6{}^{66}Zn$;

544.9694: $C_{14}H_{20}{}^{35}Cl^{37}Cl_2N_6O_6{}^{66}Zn$

546.9665: $C_{14}H_{20}{}^{37}Cl_3N_6O_6{}^{66}Zn$

Explanation for the peak at m/z = 485.1884.

Possible masses for a fragment formed from the molecular ion following the loss of two Cl coordinated to Zn and an –OH from any one Onz ligand, considering isotope distribution due to Zn [^{64}Zn (49.2%), ^{66}Zn (27.7%), ^{67}Zn (4.0%) & ^{68}Zn (18.5%)] and the two Cl atoms [^{35}Cl (75%) & ^{37}Cl (25%)].
The most probable ones based on relative abundance of Zn and Cl are shown below

485.0080: $C_{14}H_{20}{}^{35}Cl_2N_6O_5{}^{64}Zn$, 487.0049: $C_{14}H_{20}{}^{35}Cl_2N_6O_5{}^{66}Zn$

and 489.0037: $C_{14}H_{20}{}^{35}Cl_2N_6O_5{}^{68}Zn$.

Explanation for peaks with m/z = 420.0801 and 422.0779

Possible masses for a fragment formed from a molecular ion following the loss of $–CH_3$ and $–NO_2$ from each Onz ligand along with loss of a –Cl from either Onz ligand on the complex, considering isotope distribution due to the Zn [^{64}Zn (49.2%), ^{66}Zn (27.7%), ^{67}Zn (4.0%) & ^{68}Zn (18.5%)] and the Cl atom [^{35}Cl (75%) & ^{37}Cl (25%)].
The most probable ones based on relative abundance of Zn and Cl are shown below

419.9859: $C_{12}H_{19}{}^{35}Cl_3N_4O_2{}^{64}Zn$, 421.9828: $C_{12}H_{17}{}^{35}Cl_3N_4O_2{}^{66}Zn$

and 423.9816: $C_{12}H_{17}{}^{35}Cl_3N_4O_2{}^{68}Zn$

Fig. S5

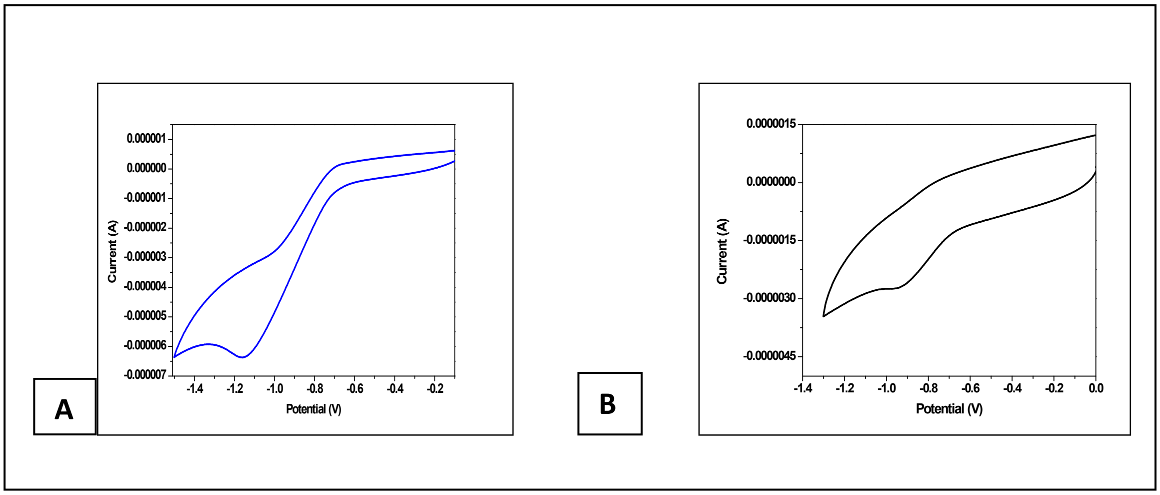

Fig. S5: Cyclic voltammograms of 1 mM $[Zn(Onz)_2Cl_2]$ in (A) 0.12 M tetrabutyl ammonium bromide in methanol and (B) in 0.12 M KCl in an aqueous solution on a glassy carbon electrode; Scan rate being 100mV/sec.

Fig. S6

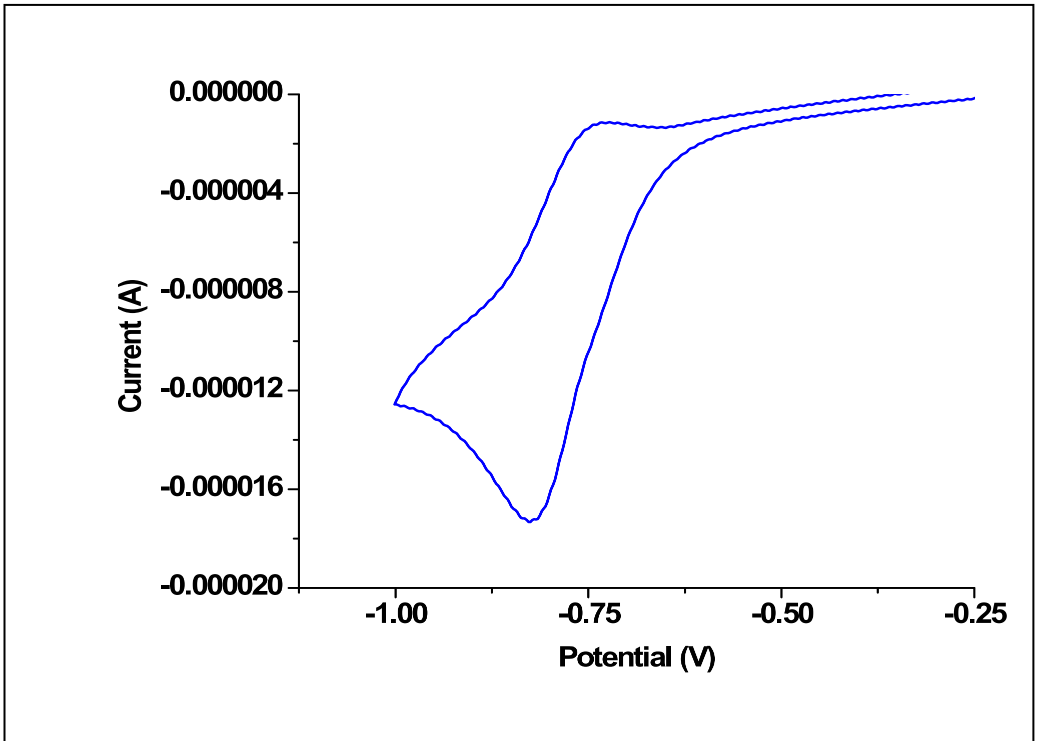

Fig. S6: Cyclic voltammogram of 1 mM Ornidazole showing a single step one electron reduction in 0.12 M KCl in an aqueous - 20% methanol solution on a glassy carbon electrode; Scan rate being 100 mV/sec.

Fig. S7

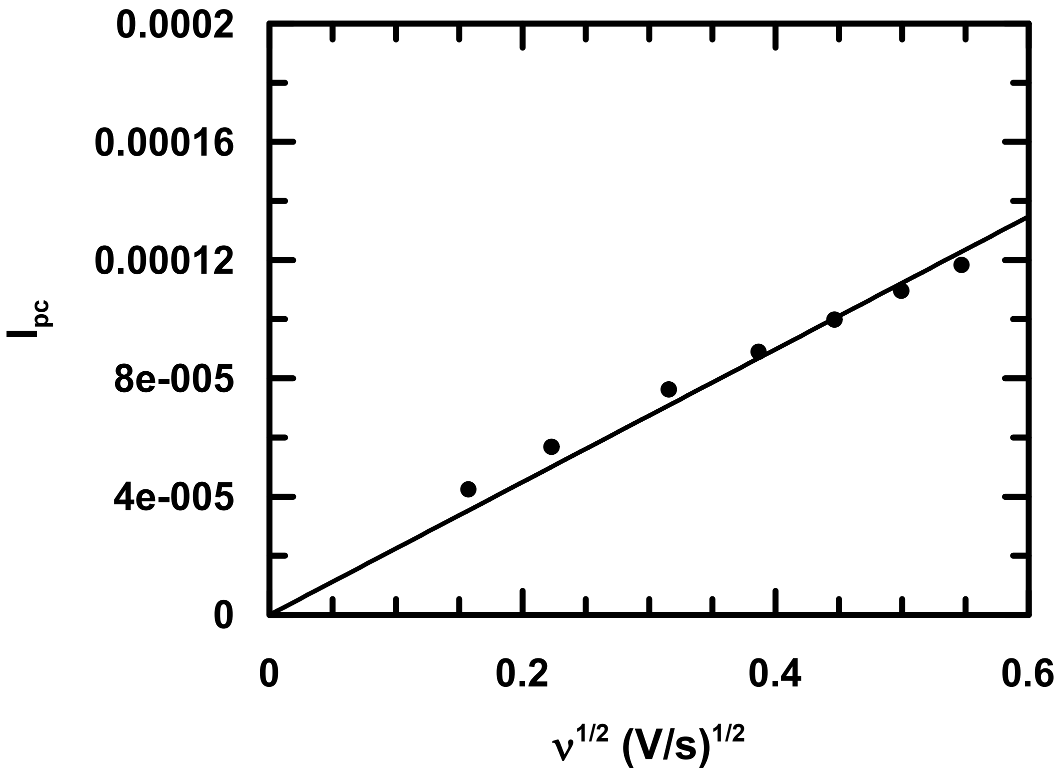

Fig. S7: Dependence of cathodic peak current on square root of scan rate for the reduction of $[Zn(Onz)_2Cl_2]$ in aqueous solution.
